# An Overview of Stress-Induced Resveratrol Synthesis in Grapes: Perspectives for Resveratrol-Enriched Grape Products

**DOI:** 10.3390/molecules22020294

**Published:** 2017-02-14

**Authors:** Md. Mohidul Hasan, Hanhong Bae

**Affiliations:** Department of Biotechnology, Yeungnam University, Gyeongsan 38541, Korea; mhasan@hstu.ac.bd

**Keywords:** resveratrol, grape, juice, wine, external stimuli, elicitation, nutraceutical

## Abstract

Resveratrol is the most important stilbene phytoalexin synthesized naturally or induced in plants, as a part of their defense mechanism. Grapes and their derivative products, including juice and wine, are the most important natural sources of resveratrol, consisting of notably higher amounts than other natural sources like peanuts. Consumption of red wine with its presence of resveratrol explained the “French Paradox”. Hence, the demand of resveratrol from grapes is increasing. Moreover, as a natural source of resveratrol, grapes became very important in the nutraceutical industry for their benefits to human health. The accumulation of resveratrol in grape skin, juice, and wine has been found to be induced by the external stimuli: microbial infection, ultrasonication (US) treatment, light-emitting diode (LED), ultra violet (UV) irradiation, elicitors or signaling compounds, macronutrients, and fungicides. Phenylalanine ammonia lyase, cinnamate-4-hydroxylase, coumaroyl-CoA ligase, and stilbene synthase play a key role in the synthesis of resveratrol. The up-regulation of those genes have the positive relationship with the elicited accumulation of resveratrol. In this review, we encapsulate the effect of different external stimuli (biotic and abiotic stresses or signaling compounds) in order to obtain the maximum accumulation of resveratrol in grape skin, leaves, juice, wine, and cell cultures.

## 1. Introduction

Consumers are very conscious and interested in their good health through controlling their diet. Their concerns are increasing due to adverse diet-related health conditions including obesity, diabetes, cardiovascular diseases, and the consequent social and economic costs [1]. Food and beverages containing phenolics have enormous health benefits, including protection against cardiovascular diseases and different types of cancer [2]. Grapes, commonly consumed fruits, contain significant amounts of phenolic compounds: stilbene, flavonols, proanthocyanidins, and anthocyanins, known to be highly effective against cardiovascular diseases [3]. Resveratrol is a stilbene compound that became more popular after the discovery of its anticancer potential, limiting tumor initiation and progression in cancer-induced rats [4]. Resveratrol was also found to induce resistance in plants against microbial infection and extend the life span in different organisms, including yeast and vertebrates [5,6]. Furthermore, phytoalexin resveratrol is a secondary metabolite related to plant defense, elicited on the infection with pathogens or due to other external stimuli.

**Figure 1:**
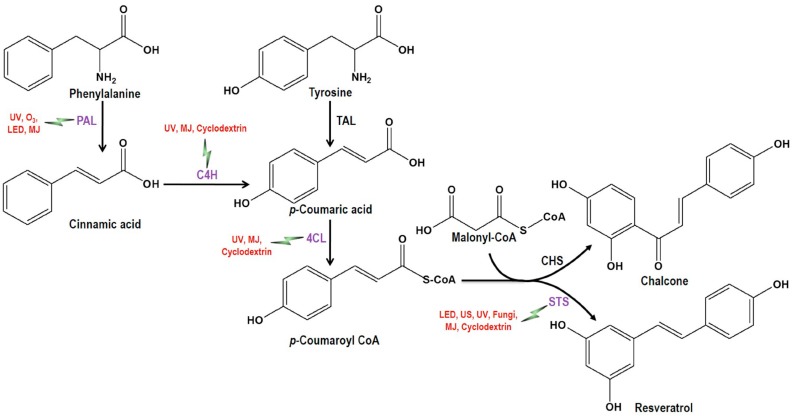
Biosynthesis pathway to resveratrol with enzymes involved and factors eliciting or inducing resveratrol synthesis (the symbol indicates 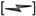 the induction of the corresponding enzymes by the mentioned elicitors or inducer). *PAL*, Phenylalanine ammonia lyase; *C4H*, Cinnamate-4-hydroxylase; *4CL*, Coumaroyl-CoA ligase; *TAL,* Tyrosine ammonia-lyase; *CHS,* Chalcone synthase; *STS*, Stilbene synthase; UV, Ultra violet; US, Ultrasonication; LED, Light-emitting diode; O_3_, Ozone; MJ = Methyl jasmonate.

Resveratrol is a polyphenol that is widely studied for its nutritional and medicinal value. The effects of different biotic and abiotic agents on the induced synthesis of resveratrol in plant tissues have also been studied widely. Resveratrol biosynthesis occurs via the phenylalanine pathway, where phenylalanine ammonia lyase (*PAL*), cinnamate-4-hydroxylase (*C4H*), coumaroyl-CoA ligase (*4CL*), and stilbene synthase (*STS*) play a core role in the synthesis (Figure 1). The end product is synthesized as *trans* form, which may isomerize to *cis* form or even transformed to *trans* and *cis*-piceid due to resveratrol 3-*O*-beta-glycosyltransferases (*O*-3-GT) [7]. *p*-coumaroyl-CoA is a product of *PAL*, which is abundant in plants and used as a precursor for the synthesis of both resveratrol and chalcone. Therefore, in stilbene-synthesizing plants, *STS* competes with chalcone synthase (*CHS*) for the synthesis of resveratrol [8]. Furthermore, stilbene synthesis pathway is the side chain of phenylpropanoid pathway, which also can be treated as an extension of the flavonoid pathway [9]. A transcriptional factor, Myb14, has been found to regulate the expression of *STS* [10].

The accumulation of resveratrol in grapes varies with the grape cultivar, genotype, location, environmental conditions, and growing season. Varying amounts of resveratrol have been reported in grape skin, seed, stem, shoot, bud, root, and leaf [11,12]. However, relatively higher amount of resveratrol can found in grape skin, whereas, less in grape juice and wine.

The global demand of resveratrol is increasing, but natural synthesis and accumulation of resveratrol are very low in grapes. Therefore, continuous efforts are ongoing to induce the accumulation of resveratrol in grape skin. Resveratrol can be induced in grapes by both biotic and abiotic factors, including fungi [13,14,15], UV-C irradiation, jasmonic acid (JA), salicylic acid (SA), H_2_O_2_, and AlCl_3_ [16,17,18,19].

Naturally, resveratrol is recovered from few plant species, including grapes. Therefore, grape and grape processed products can be considered the most relevant sources for natural as well as enhanced production of this compound. Resveratrol has been used in the nutraceutical industry and also as an anticancer and anti-aging agent. Thus, it will be worth studying the maximum induction of this compound in grapes using single or combined external stimuli. Therefore, our studies summarize the effect of different biotic and abiotic stimuli on the induction of the resveratrol accumulation in grapes. We have also summarized the effect of different external stimuli on the biosynthesis of resveratrol, regulated through an enzymatic reaction.

## 2. Elicitation of Resveratrol Due to Pathogen Infection

Usually, stilbenes are constitutively present in all parts of the grape plant but can be elicited especially in leaves and fruits. Resveratrol is found to be directly involved in vine resistance against some plant pathogens including *Botrytis cinerea* [20,21,22], *Plasmopara viticola* [13], *Rhizopus stolonifer* [23], and *Uncinula necator* [24].

In the early stages of resveratrol research, accumulation of resveratrol was detected only in injured, UV-treated, or fungi-infected leaf tissues [25]. A considerably increased amount of resveratrol (3-fold) was found around the infected leaf area of *B. cinerea*. However, the accumulated resveratrol disappeared as the disease advanced further [20,21,22]. The localized accumulation of resveratrol might help to restrict the spread of *B. cinerea* infection until the environmental conditions are unfavorable for the pathogen [22]. The presence of significant amounts of resveratrol, prior to any visible lesions in fruits, proved that the phytoalexin elicitation initiated just after recognition of the pathogen by the plant [22].

Resveratrol, in its glycosylated form, was found in higher amounts than free resveratrol in grape juice and grape skin, irrespective of the degree of *B. cinerea* infection [26]. In another study, a significantly high amount of *trans*-resveratrol was induced by the 2nd day of *B. cinerea* infection, followed by the rapid decline in the levels by the 5th day after infection [27,28]. Surprisingly, a lower concentration of resveratrol was found in wine made from botrytized grapes, as compared to the wine from healthy grapes in Germany, while slightly higher resveratrol was reported in Hungarian wines prepared from *B. cinerea* infected grapes than those from healthy grapes [29]. Recently, 4–5 times induced accumulation of resveratrol in leaves, shoots, and flowers, was noted in the blooming stage, following three days after *B. cinerea* inoculation [30] (Table 1).

Induction of resveratrol in response of *B. cinerea* is a commonly studied phenomenon. However, other fungi are also known to induce the accumulation of this compound in grapes. *P. viticola* infected grapes accumulated 5 times more resveratrol than healthy grapes, and the accumulation increased with the degree of infection [13]. Infection with *U. necator* resulted in 12 times higher accumulation of resveratrol in grape skin [14]. Unlike *B*. *cinerea*, *P. viticola* and *U*. *necator* cannot detoxify or degrade resveratrol. Hence, they are unable to suppress the production of phytoalexin in *Vitis* spp. [14]. *R. stolonifer* was also found to induce 4 to 8.5 times accumulation of resveratrol in grapes after 24 h of incubation. However, subsequent incubation resulted in gradual degradation of the accumulated resveratrol [23]. Application of mannitol, followed by supplementation of methanolic extracts of *Fusarium oxysporum* in a two-stage *Vitis vinifera* cell culture system, significantly elicited the accumulation of resveratrol [31]. A soil bacterium belonging to *Bacillus* genus, applied along with *Botrytis* conidia, was found to elicit 16 times higher accumulation of resveratrol in grape leaves, as compared to the control [27] (Table 1). In response to the pathogen attack, pathogenesis-related protein- phytoalexins noted to be synthesized in plants as a part of defense mechanism [32]. However, the underlying mechanism for the increased accumulation of resveratrol showed up-regulated expression of the *STS* gene in response to *B. cinerea* and *P. viticola* inoculation [15,33].

## 3. Accumulation of Resveratrol in Response to Light

Light is a vital element for photosynthesis of plants, ultimately affecting their growth and development, organogenesis, and production of primary and secondary metabolites [34]. Accumulation of secondary metabolites in plants, including stilbene phytoalexins, flavonoids, and carotenoids is also dependent on light [35,36] and could be enhanced by the effect of light [36,37,38,39].

LEDs as are now commonly being used as a light source in the regulation of plant growth, photomorphogenesis, enhancing quality, and production of secondary metabolites [34,40]. A recent study involving the exposure of two grape varieties, “Campbell Early” and “Kyoho”, to different light sources, showed that exposure to blue and red light caused enhanced accumulation of resveratrol in grape skin, as compared to white and purple light [41]. Blue, white, and purple light were found to increase resveratrol in Campbell Early by 8.4, 3.2, and 2.3 times, respectively, relative to the control. Similar increased accumulation of resveratrol was also found in “Kyoho” by 3.4, 3.1, and 2.1 times higher than the control with blue, white, and purple light, respectively [41] (Table 1).

Resveratrol synthesis by phenylpropanoid pathway and the induced expression of *PAL*, *CHS*, *CHI*, *STS1*, and *STS2* was observed by irradiation with different light sources [41]. The maximum increase in mRNA of *PAL* and *STS1* was observed after 24 h exposure to various light sources in Campbell Early, and the highest up-regulation of the genes was observed in response to blue light-irradiated berries [41]. In contrast, highly up-regulated expression of *PAL* and *STS1* was observed in ‘Kyoho’ grape berries irradiated with red light and picked after 48 h of treatment [41]. Light-dependent regulation of *PAL* and *STS*, the vital enzymes in the stilbene biosynthesis pathway, has also been reported previously [12,15,42].

## 4. Induced Accumulation of Resveratrol Due to Ultrasonication

Ultrasonic cleaners are used to clean a wide variety of objects by applying high-frequency sound waves to agitate materials suspended in liquids. Ultrasonic cleaners have already been designed to clean and sterilize contaminants in harvested fruits and vegetables [43]. Additionally, this technique is also applied to enhance the accumulation of secondary metabolites in plants [44]. Previously, resveratrol was found to be increased by 8–143 times over control in whole or sliced peanut kernels after ultrasonication treatment [37,45,46].

Hasan et al. applied the ultrasonication technique for the first time to increase the accumulation of resveratrol in grape skins and leaves [44]. The resveratrol accumulation was found to be increased in grape skin by 7.7 folds, after treatment with 5 min ultrasonication treatment, followed by 6 h incubation. However, the increase of resveratrol in leaves was quite less than that in grape skin. The amount of increased resveratrol in grape leaves after 15 min of ultrasonication, followed by 3 h incubation, was 1.8 fold higher than that observed in non-treated control [44] (Table 1). The accumulation of resveratrol in both grape skin and leaves was dependent on the ultrasonication time and the incubation period. A similar trend of resveratrol accumulation was shown in ultrasonication-treated peanut kernels, where 24 h of incubation resulted in higher accumulation, followed by a decrease with subsequent incubation [45].

Resveratrol-enriched grape juice was also prepared using the most effective ultrasonication treatment reported in previous work [47]. In all the grape varieties used, significantly higher elicited amount of resveratrol was found in grape juice manufactured from fruit treated with ultrasonication for 5 min, followed by 6 h of incubation [47]. Ultrasonication treatment of grape skins of Campbell Early, MBA, and Kyoho increased the resveratrol in grape juices by 1.53, 1.15, and 1.24 times, respectively [47] (Table 1).

The induction of plant secondary metabolites by ultrasonication might be due to mechanical stress, along with micro-streaming caused by acoustic cavitation [48]. It is hypothesized that ultrasonication worked through increased mass transport due to the effects of cavitations, resulting in quicker product dispersal from the enzymatic production site [49]. Semi-quantitative RT-PCR showed an increased expression level of *RS* in response to the ultrasonication treatment, where highest expression of *STS* was observed in grape skins treated with 5 min ultrasonication, followed by all subsequent incubation times up to 12 h [44]. However, in grape leaves, increased expression of *STS* was found only in 15 min of ultrasonication without further incubation, but increased after 3 h incubation, synchronizing with the observed highest accumulation of resveratrol in leaves with 15 min ultrasonication followed by 3 h incubation [44]. Phenylalanine ammonia-lyase (*PAL*) activity in response to ultrasonication treatment might also be responsible for increased accumulation of resveratrol. A dramatic increase in the *PAL* enzymatic activity was observed in *Panax ginseng* cells due to the ultrasonication treatment [50], which is accountable for deamination of phenylalanine coumaryl CoA, the precursor of resveratrol biosynthesis [51].

## 5. Accumulation of Resveratrol Due to Signaling Chemicals

Among the stilbenes, resveratrol is the prime phytoalexin in plants, produced in response to external stimuli including UV and different signaling chemicals. For the induction of plant secondary metabolites related to the plant defense system, JA plays a key role in the signal transduction pathway [52,53].

Notable induction of stilbene accumulation with the addition of methyl jasmonate (MJ; the JA derivative) at day 6 after starting the culture was reported [54]. MJ was found to cause a 3-fold elicitation of *trans*-resveratrol, following 18 h incubation, whereas MJ and sucrose together elicited the accumulation of *trans*-resveratrol by 6-fold after 6 h of incubation. In addition, MJ/sucrose treatment was found to produce 2-fold extra-cellular yield of resveratrol than MJ alone [55]. In a two-stage culture system, JA, along with mannitol, was found to maximize the accumulation (10.5 times) of resveratrol in callus culture because of synergistically enhanced metabolic production [31] (Table 1). However, the induction of resveratrol with JA was found to be consistent with the up-regulated expression of *PAL* and *STS*, showing the transcriptional control of the synthesis of resveratrol [55].

Cyclodextrins, alone or in combination with MJ, were found to increase the extracellular accumulation (3- to 20-fold) of resveratrol in grapevine cell suspensions. However, the elicited accumulation was higher with MJ than that with cyclodextrins [56,57] (Table 1). The *STS* expression was found to be higher after 24 h of cyclodextrins treatment, whereas MJ incubated cells showed stable higher expression after 72 h. Moreover, *PAL*, *C4H*, *4CL*, *CHS* of the anthocyanins/isoflavonoids pathway were found to be stimulated by both the elicitors [56].

**Figure 2:**
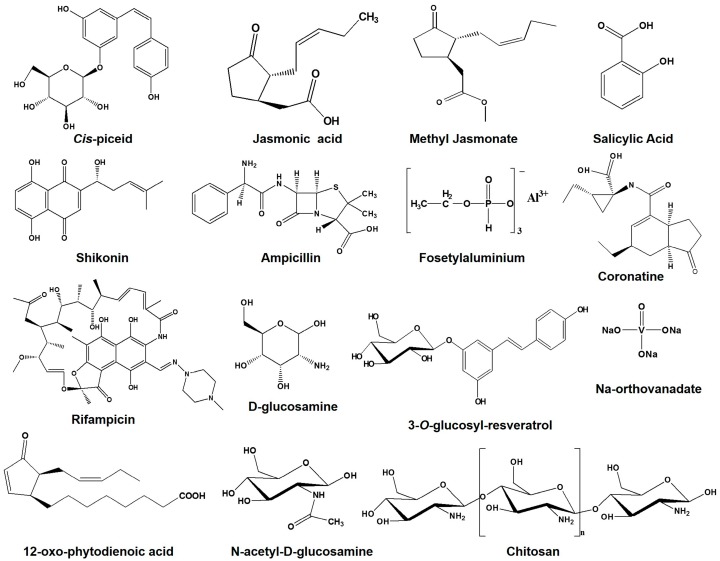
Molecular structure of different compounds or elicitors mentioned in this review.

In another cell suspension culture, elicited accumulation of intracellular resveratrol with JA, SA, glucan (GLU), and chitosan (CHI) were found to be manifold, where the combined treatment of JA and GLU induced the extracellular accumulation of resveratrol by 10-fold (Figure 2) [58]. However, an industrial scale increased production of resveratrol (2400 mg/L) was obtained by the combined treatment of Amberlite XAD-7 resin, JA, and GLU [58]. SA was found to induce the resveratrol accumulation alone (2 times), but a negative effect of the combined use of SA and JA was observed on the accumulation of resveratrol, verifying the inhibitory effect of SA on the synthesis and signal transduction of JA [59] (Table 1).

JA and coronatine (COR) were found to show an analogous increased accumulation style of resveratrol at 48 h and 96 h (1600 and 1900 μg/g DW), whereas 12-oxo-phytodienoic acid (OPDA) elicited stable amounts of resveratrol (2200 at 48 h μg/g DW). However, MJ was found to accumulate 40%, 50%, and 60% more resveratrol over JA, COR, and OPDA, respectively [60] (Table 1, Figure 2). The maximum resveratrol synthesis and secretion in culture media were observed in cell suspension due to the synergistic activities of cyclodextrins and COR combined treatment, as compared to the individual treatment [61]. The *PAL* and *STS* genes were found to be highly expressed with COR. However, the induced amount of resveratrol was not detected in a spent medium, demonstrating that post-transcriptional or post-translational behavior may also regulate the expression of the genes [61].

A chitin derivative CHI was also found to increase the accumulation of intracellular resveratrol by 10.5-fold, which further increased with increasing concentration of CHI [58,62]. In suspension culture of *Vitis vinifera* L. cv. Gamay Freaux, indanoyl-isoleucine, *N*-linolenoyl-l-glutamine, and insect saliva (from Manduca sexta larvae) considerably increased the accumulation of phenolic acids, especially 3-*O*-glucosyl-resveratrol, by 7.0-fold, relative to the control, after 24 h of treatment, especially with saliva [63]. On the contrary, cell cultures of *V. vinifera* (Barbera) treated with MJ, JA, SA, CHI, *N*-acetyl-d-glucosamine, Na-orthovanadate, d-glucosamine, ampicillin, and rifampicin (Figure 2) were found to increase the level of resveratrol [64,65]. Similarly, MJ, CHI, and yeast extracts were also found to increase the accumulation of phenolic content in grape and wine [66]. Yeast cell contains b-1,3- and b-1,6-glucans, mannoproteins, lipid, sterol, and chitin where many of them act as elicitors along with triggering of plant defense [67]. However, accumulation of plant secondary metabolites and up-regulation of *PAL* in cell cultures treated with yeast extracts justified its effects on resveratrol accumulation [68]. However, CHI alone was found to increase 63% endogenous accumulation of resveratrol after 4 days, along with the decrease of resveratrol release in culture media, as compared with the control [69] (Table 1). How CHI increased the accumulation of resveratrol is yet to be clearly understood. CHI was found to promote the stilbene pathway enzyme proteins, which can induce the de novo biosynthesis of resveratrol. In addition, the accumulation of resveratrol also depends on the concentration, molecular weight and degree of *N*-acetylation of CHI [69,70]. CHI was also found to induce the accumulation of six different STS protein spots in comparison with the control, whereas *STS* transcript was not observed in the northern blot analysis, ensuring that the up-regulation of *STS* occurred at the post-translational level and can induce PR proteins including chitinase, glucanase, and *PAL* in grapes [69,71,72].

## 6. Induction of Resveratrol by UV Irradiation 

UV-C light range from 200 to 280 nm wavelength is a germicidal, non-ionizing radiation that has been extensively using to sterilize fresh fruits and vegetables to maintain their quality [73,74]. UV-C is also very popular in the field of enhancing the production of the stilbene resveratrol in grape berries and grape process products, including grape juice and wine.

Previously, the accumulation of resveratrol was undetectable or less detectable in healthy leaves of the grape vine, whereas 50–400 μg/g (fresh weight, FW) elicited amount of resveratrol was recorded in UV (260–270 nm) irradiated grape berries [23,25,75,76]. A long time (10–15 min) exposure with short distance (15 cm) of UV-C irradiation source from the grape leaf was found to increase resveratrol accumulation from not detectable to 750 μg/g (FW) resveratrol, which persisted for the subsequent 2 days [77,78,79]. Mature Napoleon grapes treated with UV-B and UV-C for 30 min and stored at 0 °C for 10 days, followed by incubation at 15 °C for 5 days, induced the resveratrol accumulation by 3 and 2 times, respectively, whereas UV-C treatment alone was found to increase the accumulation in immature grape [80]. In an unripe shipment, Californian table grape showed 4 times increased accumulation of resveratrol due to UV irradiation, whereas degradation occurred with fully matured grapes [81]. Similarly, increased accumulation (15.2 μg/g FW) of resveratrol was observed in young cluster of berries irradiated with UV-C for 6 min, followed by 24 h of incubation [82] (Table 1).

However, for higher accumulation of resveratrol, an optimization of UV irradiation treatment was done and also patented under the following conditions: distance of 40 cm, irradiation time of 30 s, source power of 500 W, and storage time of 3 days; this resulted in 3.4 times higher resveratrol in Flame cultivar and 2315 times in Red Globe cultivar, compared to the untreated controls [83]. With the optimized protocol, increased concentrations (2.5 and 2 times) of resveratrol in grape, juice, and wine, respectively, were also observed [84,85] (Table 1).

Another optimization of UV-C irradiation was done and patented regarding the increased accumulation of resveratrol in fruit and vegetables with irradiation strength ranging from 30–510 W for less than 1 min, followed by incubation in 2–4 days, resulting in 10.8-fold higher accumulation than that observed in the untreated control [18,86,87,88]. UV treatment for 610.2 mJ/cm^2^, followed by storage at 0 °C resulted in increased accumulation of resveratrol by 7–8 times after 1 and 6 days in Gerbong grapes, respectively [89,90] (Table 1).

UV-C irradiation, following CHI application in grape berry, was found to be more effective in inducing the accumulation of resveratrol (23.2 times) than UV irradiation alone (18.1 times), while CHI alone did not induce an accumulation of resveratrol [72]. UV-C irradiation of *Botrytis cinerea* inoculated grape berries was found to decrease the infection and lesion size due to the induction of resveratrol [91].

Appropriate maceration conditions (2 h at 45 °C with 0.2% sodium metabisulfite (Na_2_S_2_O_5_) are urgent for the proper extraction and solubilization of the induced stilbene in grape juice and UV-C treated grape variety. Grape variety Superior, following proper maceration, resulted in 35 times more accumulations of resveratrol in juice without any negative impact on sensory properties [92] (Table 1).

A UV-B laser (302.1 nm, ionizing wavelength) treatment for 45 min was found to induce 6-fold resveratrol, immediately after the irradiation, whereas 300 nm radiation (non-ionizing wavelength) resulted in negligible accumulation [93]. Previous studies showed that single UV-C photon irradiation was responsible for elicited biosynthesis of resveratrol by *STS* activity [94]. However, the maximum biosynthesis of resveratrol was reported in response to UV-irradiation in 260–270 nm, signifying that DNA acts as a photoreceptor and the phenylalanine-polymalonate pathway is the key to resveratrol biosynthesis [25]. However, a strong elicitation of resveratrol at 302.1 nm further demonstrates that two-photon absorption could offer sufficient energy for the activation of phenylalanine-polymalonate pathway instead of single photon excitation [95].

Induced accumulation of resveratrol in grape skin, in accordance with the expression of genes related to stilbene synthase (*STS*), phenylalanine ammonia-lyase (*PAL*), and chalcone synthase (*CHS*), was higher in Muscat Bailey A (1910 µg/g FW) after 8 h of UV-C irradiation, as compared to the accumulation in other two varieties tested [96] (Table 1). A stage and time-dependent increase in *STS* protein over the course of 0–96 h after UV treatment was reported, where *STS* expression was induced within 6 h and reached a maximum after 18 h in 30 days after fruiting, DAF. Contrarily, maximum STS proteins were observed after 18, 48, and 96 h of UV treatment of 70, 90, and 120 DAF, respectively [12]. *STS* was found to be distributed in organs and tissues specifically, and the induction of resveratrol by UV-C was controlled at the transcriptional and translational level [12]. However, the increase in STS enzyme in response to UV was not only dependent on time but also dependent on the developmental stage of berries and incubation after UV irradiation. Moreover, the elicited STS enzymes are mostly present on the grape skin cell wall, secondary cell wall, and in minimum amounts in the chloroplast [97]. In addition, the expression of *STS* genes was found to be higher before veraison, followed by a decrease up to maturity [98]. A total of 22 *STS* genes showed increased expression following the treatment with UV-C, and *VaSTS* expression was revealed as an important factor for the accumulation of resveratrol. Up-regulation of *PAL* with *C4H* and *4CL* was found in response to UV-C treatment, along with the down-regulation of *CHS* [99,100], proving that the competitive relationship between *STS* and *CHS*, in response to UV-C, may play a vital role in the accumulation of resveratrol in grape berries [90].

## 7. Elicitation of Resveratrol Due to Ozone

Ozone extends the shelf life of table grapes by preventing fruit decay, where it can be applied as pre-storage or storage room treatment in the form of fumigation or as ozonated water [101]. In addition, ozone is also found to stimulate the synthesis of resveratrol in storage conditions but not in consistent amount. In order to control fungal attack in storage, fumigation of ozone was applied and has been reported an increase of resveratrol level in grape berry skin comparable with UV-induced amount [102]. Comparable accumulation of resveratrol (1250 mg/100 g FW) was also found with 3.88 g/h treatment for 5 h and storage for 2 days, where the total stilbenoids produced were higher than those from the UV treatment [18]. By maintaining the standard atmospheric condition in storage at 15 °C and application of 8 ppm ozone, around 3.14 times elicited accumulation of resveratrol over control was found in cv. Napoleon table grapes [103]. Later, with an intermittent ozone (2 ppm) treatment, rather than continuous treatment, for 12 h/day was found to increase the accumulation of resveratrol by 17%–55% over control, along with the reduction in the decay of stored grapes [104]. Further, elicited accumulation of resveratrol in *V. vinifera* callus cultures from the leaf with the fumigation of ozone was obtained after 24 h of the treatment [105] (Table 1). Increased *PAL* activity and a rapid induction of parts of the grapevine *STS* promoter, together with a P-GUS reporter gene in the transgenic tobacco plant, was observed after treatment with ozone (0.1 µL/L, 12 h), revealing the underlying mechanism of the resveratrol induction with ozone [105,106]. In the literature, there is little information on the production of resveratrol in response to ozone treatments directly on the plant. It has been recently published that resveratrol was not detected in the leaves of two cultivars San Giuseppe and Maturano after ozone fumigation [107]. Previously, a lower concentration of ozone in the form of ozonated water was found to act against microbial growth but did not induce the accumulation of resveratrol in stored grapes [18,108]. However, a higher ratio of ozone concentration to exposure time is needed for the induction of resveratrol, which might cause the reduction of the sensory properties of grape berries. Therefore, using this approach could be suitable for the production of resveratrol-enriched products rather than elicitation of resveratrol in table grapes.

## 8. Induction of Resveratrol Due to Metal Salt

Metallic salt acts as a signaling compound and salts of mercury or copper were found to induce the production of isoflavanoids, sesquiterpenes, and also stilbene in *Veratrum grandiflorum* [109]. A systemic fungicide “fosetylaluminum” contains aluminum chloride (AlCl_3_, Figure 2) was used against downy mildew disease of grape to induce de novo synthesis of phytoalexin, and following 15 h of incubation, leaves showed bright fluorescence under UV (254 and 366 nm) light, a typical characteristic of *trans*-resveratrol [25]. The fluorescence was detected (presence of resveratrol) in principal veins, followed by fine venation, and ultimately covered both the surface of the leaf representing the systemic nature of aluminum chloride. The abaxial surface was found to show fluorescence only after UV treatment for the synthesis of resveratrol [25,110]. All concentrations of AlCl_3_ (from 7 to 90 mM) were found to have the potential to elicit the amount of resveratrol in the leaves of *Vitis rupestris*, where marginal concentrations (7 mM) were needed to obtain a phytoalexin signal in leaves [111]. Similarly, induced accumulation of resveratrol in grape leaves (5.2 times) and berry skin (1.5) were observed with CaCl_2_, where UV irradiation, in combination with CaCl_2_, accumulated 37.2 times higher resveratrol [19] (Table 1). Furthermore, inefficient or poorly efficient stilbene synthase gene expression with an application of AlCl_3_ and CaCl_2_ individually were found in relation to the induced accumulation of resveratrol [19,94]. UV irradiation combined with CaCl_2_ showed significant up-regulation of *PAL*, *4CL*, *CH4*, and *STS*, parallel with the resveratrol accumulation, suggesting that Ca^2+^ may participate in the signal transduction pathway of UV-C-induced biosynthesis [19]. Pea treated with CdCl_2_ has been hypothesized to demonstrate a definite role of metallic salt in inducing the synthesis of phytoalexin [112]. 

## 9. Induced Accumulation of Resveratrol Due to Pesticides

Plant diseases are the vital reasons for the reduced yield of crops. Hence, farmers need to apply fungicides or pesticides to protect their crops. Phenolics, including resveratrol, were found to be induced by microbial infection, as well as UV irradiation [83]. Wine from cv. Campania treated with wettable sulfur (a fungicide and insecticide) showed an increased accumulation of *trans*-resveratrol by 1.6 times, as compared with the control [113]. Grapes treated with other pesticides (Quinoxyfen, Fenarimol, Penconazole, Dinocap) also resulted in higher amounts of *trans*-resveratrol accumulation over control [113]. The wines prepared from wettable sulfur treated grapes showed the highest amount (1.4 times) of *trans*-resveratrol, relative to the control [114] (Table 1). Furthermore, the wines showed varying degrees of resveratrol, resulting from several factors influencing the wine composition: vinification process (red or white), climatic conditions, different agronomical practices, and exposure of the vineyards to different atmospheric agents.

## 10. Effect of Enological Practice for the Increased Amount of Resveratrol

Grape skin shows high accumulation of resveratrol. Hence, maximum extraction of resveratrol occurred with long fermentation time [22]. Different processing techniques including maceration have great impacts on the improved extraction of resveratrol, ultimately leading to the increased resveratrol concentration in grape juice and wine. Maceration was found to increase the extraction of resveratrol in wines by 10-fold compared with just pressing a little for a very short time on grape skins. On the contrary, macerated wines contain up to 13-fold more resveratrol than non-macerated wines [22,115,116,117] (Table 1). Likewise, the maximum accumulation of resveratrol was also found in Merlot wine M12 up to 10 days of maceration with French Yeast and SO_2_ [117]. In addition, maceration time and type of yeast and concentration of SO_2_ were also found to be important factors influencing the concentration of resveratrol in wine [117]. 

## 11. Induction of Resveratrol Due to Fertilizer

Mineral nutrition not only affects the yield and quality of the plant but also plays a significant role in the resistance or susceptibility to disease by interfering with the biochemical mechanism. Nitrogen is an important nutrient for the synthesis of plant secondary metabolites such as alkaloids, anthocyanins, and shikonin (Figure 2) from cell suspension cultures [118]. Earlier, application of nitrogen in a hybrid grape variety Ga-58-30 and Fr 993-60 showed increased resistance to powdery and downy mildew disease with the elicited amount (4970 s.u.) of resveratrol in leaves [119]. Furthermore, increased accumulation of resveratrol was observed with high potassium, along with low levels of nitrogen, where high potassium levels could not diminish the negative effect of high level of nitrogen [120]. The basis of defense mechanism or the production of phytoalexin evolved at a low nitrogen level for the equilibrium between primary and secondary pathway, ensuring the movement of a large pool of the phenolics and alkaloids to the shikimate pathway [121]. For these reasons, nitrogen application in the vineyard has to be judicious in order to obtain a high yield of grapes, along with a maximum accumulation of resveratrol. A synchronized application of nitrogen with proper vegetative growth could be a better way to resolve the incompatible relationship of increased level of nitrogen with an accumulation of resveratrol [122]. However, harvested grapes kept for 6–15 h at room temperature in a vacuum chamber packed with nitrogen showed increased accumulation of resveratrol [123]. Recently, ammonium nitrate NH_4_NO_3_ as a source of nitrogen was found to elicit the accumulation of resveratrol at 14th day by 5.6-fold in cell culture [124] (Table 1).

## 12. Conclusions and Future Prospects

Consumers around the world are showing more interest in using natural herbal products instead of synthetic ones to treat various diseases. The demand of resveratrol is increasing due to its potential role in cardiovascular diseases, anti-cancer effects, and anti-aging effects. To meet the increasing demand, currently, resveratrol is marketed in the form of herbal or dietary complement as capsules, powders, and pills. However, consumers and nutritionists are continuously searching for the better supplements from natural sources in order to reduce the negative effect on human health. Most of the available resveratrol-enriched foodstuffs have originated from botanical sources. Along with resveratrol, botanical sources also provide derivatives, cofactors, and different phytonutrients, which may give the synergistic effect of the nutraceutical products. Several strategies for the biotic synthesis of resveratrol, including engineering of yeast, bacteria, or recombinant plants have been tried to ensure the consistent supply of resveratrol and its derivatives. However, continuous efforts are being made to find out better sources or strategies for the production of the higher amount of resveratrol. As grape skin is the most important source of resveratrol in red wine and juice, elicitation of resveratrol accumulation in grape skin can have practical applications. Hence, we summarize efforts to increase the accumulation of resveratrol in grapes.

Few plant species, including grape, peanuts, pine tree, and tomato produces resveratrol. However, grape contains more resveratrol than all other natural sources. Almost all the plant parts of grape including grape skin, stem, leaf, petiole, and root contain resveratrol. However, the maximum resveratrol is present in grape skin. As the major findings, UV treatment significantly increased the accumulation of resveratrol more than 2000-fold. Recently, US and LED exposure markedly induced the amount of resveratrol for about 8-fold in grape skin, which helped to produce resveratrol-enriched grape juice and wine. In addition, the combined treatment of UV with CHI, or other elicitors induced higher resveratrol concentration than by UV treatment alone. The amount of resveratrol-induced in cell suspension cultures, varied in response to different elicitors such as JA, SA, and COR. However, to meet the increasing demand of this compound, more effort should be taken to elicit higher accumulation of resveratrol in natural sources. To attain maximum level of resveratrol content, the application of various biotechnological strategies is important. Simultaneously, continuous searching of effective and economically viable elicitors and genes for microbes or plant transformation are also needed. 

Currently, nutraceutical is the most promising market for resveratrol. As a food supplement, metabolic engineering of resveratrol in microbes could be the most effective way to meet the increasing demand. Resveratrol in the grape skin is induced by different external factors and might be the most important source to meet the increasing demand. Ultrasonication cleaner, along with tap water, is usually used to clean many fruits and vegetable to produce contamination-free produce along with induction of resveratrol in grapes and peanut [43,47]. US, along with UV and/or LED could be a significant way to increase the accumulation of resveratrol in grape skin. However, induced accumulation of resveratrol in the grape skin might have a significant practical implication in the grape juice and red wine industry.

Phytochemicals in plants can be abruptly increased by the genetic transformation of the plant with important genes [125] and could be a possible way to increase the accumulation of secondary metabolites, including resveratrol [125]. Furthermore, selection of bacterial strain could be the most important criterion for successful and practical applications of plant transformation techniques in order to accumulate maximum resveratrol.

Application of different combinations of elicitors known as inducers of plant secondary metabolites could be another strategy for the increased production of resveratrol in grape skin or in cell suspension culture along with US, LED, and/or UV. However, prior to the application of hormones or elicitors, their toxicological effect on human health should be tested. Moreover, establishing routine cell or tissue suspension cultures, selection of high-producing cell lines, and optimization of culture media could be a better route to enhanced resveratrol production.

## Figures and Tables

**Table 1:** Induced amount of resveratrol in grape skin, leaves, juice, wine, and cell/callus cultures by different external stimuli.

Eliciting Agent		Induction of Resveratrol up to (by Fold/Amount)					References
		Grape	Leaf	Juice	Wine	Cell/Callus Cultures	
Biotic agents	*B. cinerea*	3.2	5	-	little	-	[20,21,22,26,27,28,29,30]
	*P. viticola*	5	-	-	-	-	[13]
	*U. necator*	12	-	-	-	-	[14]
	*R. stolonifer*	8.5	-	-	-	-	[23]
	*F. oxysporum* + mannitol	-	-	-	-	6	[31]
	*Bacillus +* *Botrytis*	-	16	-	-	-	[27]
Abiotic agent/Physical force	LED	8.4	-	-	-	-	[41]
	US	7.7	1.8	1.53	-	-	[44,47]
	UV	2315	750	35	2	2	[18,23,25,75,76,77,78,79,80,81,82,83,84,85,86,87,88,89,90,92,93,94,95,96]
	UV + CHI	23.2	-	-	-	-	[72]
	UV + CaCl_2_	16.6	37.2	-	-	-	[19]
	Ozonization	3.14	-	-	-	1.5	[18,101,102,103,104,105]
Elicitors/signaling molecules	JA/MJ	-	-	-	-	20	[54,55,57,58,64,65,66]
	JA + Mannitol	-	-	-	-	10.5	[31]
	MJ + Sucrose	-	-	-	-	6	[55]
	MJ + GLU	-	-	-	-	10	[58,66]
	SA	-	-	-	-	2	[58,59,64,66]
	Manduca sexta larvae	-	-	-	-	7	[63]
	Cyclodextrins	-	-	-	-	3	[57]
	Cyclodextrins + MJ	-	-	-	-	>20	[56]
	Amberlite XAD-7 + JA + GLU	-	-	-	-	2400 µg/g dw	[58]
	COR	-	-	-	-	1900 µg/g dw	[60]
	OPDA	-	-	-	-	2200 µg/g dw	[60]
	CHI	-	-	-	-	63%	[58,62,64,66,69]
	AlCl_3_ (Fosetylaluminum)	-	350	-	-	-	[110]
	CaCl_2_	1.5	5.2	-	-	-	[19]
Fertilizer	Nitrogen	-	5.6	-	-	-	[118,121,123]
	Potassium + Nitrogen	-	>2	-	-	-	[119]
Fungicides	Wettable sulpher	-	-	-	1.6	-	[112,113]
	Quinoxyfen, Fenarimol, Penconazole, Dinocap	-	-	-	2.25	-	[112]
Others	Enological practice	-	-	13	-	-	[22,114,115]
	Maceration	-	-	-	10	-	[22,114,115,116]

## Abbreviations:

US	Ultrasonication
UV	Ultra violet
LED	Light-emitting diode
*PAL*	Phenylalanine ammonia lyase
*STS*	Stilbene synthase
*RS*	Resveratrol synthase (Syn. *STS*)
*C4H*	Cinnamate-4-hydroxylase
*4CL*	Coumaroyl-CoA ligase
*CHS*	Chalcone synthase
*TAL*	Tyrosine ammonia-lyase
*STS1*	Stilbene synthase1
*STS2*	Stilbene synthase2
*O*-3-GT	3-*O*-beta-glycosyltransferases
JA	Jasmonic acid
SA	Salicylic acid
CHI	Chitosan
MJ	Methyl jasmonate
GLU	Glucan
COR	Coronatine
OPDA	12-oxo-phytodienoic acid
DAF	Days after fruiting
AlCl_3_	Aluminum chloride
CaCl_2_	Calcium chloride
CdCl_2_	Cadmium chloride
Na_2_S_2_O_5_	Sodium metabisulfite
FW	Fresh Weight
